# Supplementary material for: The Emergence of New Aggressive Leaf Rust Races with the Potential to Supplant the Resistance of Wheat Cultivars
Source: Biology (Basel). 2021 Sep 16;10(9):925. doi: 10.3390/biology10090925 (PMC8471702; doi:10.3390/biology10090925)
Supplement: Supplementary file 1 [file biology-10-00925-s001.zip › biology-1357887-supplementary.pdf]

Supplementary materials

Table S1. Pt-code for the 20 differential hosts of *Puccinia triticina* in ordered sets of four and additional set five.

		Infection type produced on near isogenic Lr lines			
Pt-code[a]	**Host set 1:**	1	2a	2c	3
	**Host set 2:**	9	16	24	26
	**Host set 3:**	3ka	11	17	30
	**Host set 4:**	10	18	21	2b
	**Host set 5:**	14b	15	36	42
**B**		Low	Low	Low	Low
**C**		Low	Low	Low	High
**D**		Low	Low	High	Low
**F**		Low	Low	High	High
**G**		Low	High	Low	Low
**H**		Low	High	Low	High
**J**		Low	High	High	Low
**K**		Low	High	High	High
**L**		High	Low	Low	Low
**M**		High	Low	Low	High
**N**		High	Low	High	Low
**P**		High	Low	High	High
**Q**		High	High	Low	Low
**R**		High	High	Low	High
**S**		High	High	High	Low
**T**		High	High	High	High

[a]Pt-code consists of the designation for set 1 followed by that for set 2, etc. L = Low infection type (avirulent pathogen); H = High infection type (virulent pathogen).

Table S2. Wheat leaf rust infection types used in disease assessment for seedling stage according to Johnston and Browder [31].

Host response (class)	Infection type	Disease symptoms
**Immune**	0	No uredia or other macroscopic sign of infection.
**Nearly immune**	0;	No uredia but hypersensitive necrotic or chlorotic flecks present.
**Very resistant**	1	Small uredia surrounded by necrosis.
**Moderately resistant**	2	Small to medium uredia surrounded by chlorosis or necrosis.
**Moderately susceptible**	3	Medium-sized uredia that may be associated with chlorosis.
**Very susceptible**	4	Large uredia without chlorosis or necrosis or rarely necrosis.
**Heterogeneous**	X	Random distribution of different variable sized uredia on single leaf.

Table S3. Code, nucleotide sequence and G+C (%) of primers used in the random amplified polymorphic DNA (RAPD) reactions.

Primer	Sequence	(G+C) %
1	5′- GGTGCGGGAA- 3′	70
2	5′- GTTTCGCTCC- 3′	60
3	5′- GTAGACCCGT- 3′	60

Table S4. The pedigree list of the monogenic lines (*Lr* genes) used in this study.

No.	*Lr* gene	Origin of seed resource
**1**	*Lr1*	TC*6/Centenatrio (RL6003)
**2**	*Lr2a*	TC*6/Webster (RL6016)
**3**	*Lr2b*	TC*6/Carine (RL6016)
**4**	*Lr2c*	TC*6/Lorous (RL6047)
**5**	*Lr3*	TC*6/Democrat (RL6002
**6**	*Lr3ka*	Bage/TC*6 (RL6042)
**7**	*Lr9*	Transefer/8*TC (RL6010)
**8**	*Lr10*	TC*6/Exchange (RL6004)
**9**	*Lr11*	Kussar (W976)
**10**	*Lr14b*	TC*6/Maria Escobar (RL6006)
**11**	*Lr15*	TC*6/Kenya 1483 (RL6052)
**12**	*Lr16*	TC*6/Exchange (RL6005)
**13**	*Lr17*	Klein Lucero/6*TC (RL6008)
**14**	*Lr18*	TC*6/Africa 43 (RL6009)
**15**	*Lr19*	TC*7/Tr (RL6040)
**16**	*Lr21*	TC*6/RL5406 (RL6043)
**17**	*Lr24*	TC*6/Agent (RL6064)
**18**	*Lr26*	TC*6/St-1-25 (RL6078)
**19**	*Lr28*	CS2D-2M
**20**	*Lr30*	TC*6/Terenz10(RL6049)
**21**	*Lr36*	E84018
**22**	*Lr42*	*T. tauchii*

Table S5. Primer names, sequences, annealing temperatures and references from *Lr* genes associated markers used in this study.

Gene	Name	Primer sequences (5'-3')	Annealing temperatures
Lr19	SCS73719-1<br>SCS73719-2	TCG TCC AGA TCA GAA TGT G<br>CTC GTCGATTAGCAGTGAG	55
Lr28	*Lr* 28-01	CCC GGC ATA AGT CTA TGG TT	50
	Lr 28-02	CAA TGA ATG AGA TAC GTG AA	
